# A Proteomics Data Mining Strategy for the Identification of Quinoa Grain Proteins with Potential Immunonutritional Bioactivities

**DOI:** 10.3390/foods12020390

**Published:** 2023-01-13

**Authors:** Rocío Galindo-Luján, Laura Pont, Victoria Sanz-Nebot, Fernando Benavente

**Affiliations:** 1 Department of Chemical Engineering and Analytical Chemistry, Institute for Research on Nutrition and Food Safety (INSA·UB), University of Barcelona, 08028 Barcelona, Spain; 2 Serra Húnter Programe, Generalitat de Catalunya, 08007 Barcelona, Spain

**Keywords:** antimicrobial peptides (AMPs), bioactivity, data mining, immunonutrition, oxidative stress induced peptides (OSIPs), proteomics, quinoa, serine-type protease inhibitors (STPIs)

## Abstract

Quinoa proteins are attracting global interest for their wide amino acid profile and as a promising source for the development of biomedical treatments, including those against immune-mediated diseases. However, information about the bioactivity of quinoa proteins is scarce. In this study, a quinoa grain proteome map obtained by label-free mass spectrometry-based shotgun proteomics was investigated for the identification of quinoa grain proteins with potential immunonutritional bioactivities, including those related to cancer. After carefully examining the sequence similarities of the 1211 identified quinoa grain proteins against already described bioactive proteins from other plant organisms, 71, 48, and 3 of them were classified as antimicrobial peptides (AMPs), oxidative stress induced peptides (OSIPs), and serine-type protease inhibitors (STPIs), respectively, suggesting their potential as immunomodulatory, anti-inflammatory, and anticancer agents. In addition, data interpretation using Venn diagrams, heat maps, and scatterplots revealed proteome similarities and differences with respect to the AMPs, OSIPs, and STPIs, and the most relevant bioactive proteins in the predominant commercial quinoa grains (i.e., black, red, white (from Peru), and royal (white from Bolivia)). The presented proteomics data mining strategy allows easy screening for potentially relevant quinoa grain proteins and commercial classes for immunonutrition, as a basis for future bioactivity testing.

## 1. Introduction

In recent decades, several diseases have appeared due to declining immunity, which is directly related to lifestyle factors such as physical activity, sleep, stress, and especially, dietary habits [1,2]. Nowadays, one approach to modulate immune-mediated diseases, including cancer [3], consists of incorporating immunomodulatory and anti-inflammatory nutrients into the diet (i.e., immunonutrition) [4], such as those provided by plant-derived bioactive proteins and the peptides resulting from their hydrolysis [5,6,7]. Depending on their bioavailability, these peptides could resist the action of digestive enzymes during their transit through the gastrointestinal tract and cross the intestinal epithelial barrier, reaching the target organs intact and exerting their health-promoting effects [8]. Among the bioactive proteins and peptides with potential immunonutritional bioactivities, antimicrobial peptides (AMPs), oxidative stress induced peptides (OSIPs), and serine-type protease inhibitors (STPIs) are attracting the greatest attention [9,10,11].

AMPs are natural products found across diverse taxa as part of the innate immune system against pathogen attacks. AMPs are structurally and biochemically highly diverse and they mainly present antimicrobial, immune-regulatory, and anti-inflammatory activities [12,13,14,15]. In addition, some AMPs can exhibit cytotoxic and anticancer activities against different cancer cells [9]. Specifically, from the 12 plant-derived AMP families that are described to date in the literature, 3 of them present AMPs with cytotoxic and anticancer bioactivities, i.e., defensins, thionins, and cyclotides [9]. OSIPs are responsible for modulating oxidative stress signaling pathways mediated by reactive oxygen species (ROS), increased levels of which can favor carcinogenesis, cardiovascular diseases, neurological disorders, and chronic inflammation [10,16,17]. In contrast to plant-derived AMPs, which have been extensively described for a wide variety of organisms [9,15], OSIPs still remain relatively unknown, with *Arabidopsis thaliana* being the most widely studied plant source of bioactive peptides involved in oxidative stress tolerance [10,18]. Finally, STPIs, in addition to being effective against cardiovascular, inflammatory diseases, and neurological disorders, have been connected with immunomodulation and cancer prevention [11,19]. Specifically, plant-derived STPIs have been described as being involved in the prevention and treatment of hepatocarcinoma, which can be caused by dietary habits that result in hepatic immunometabolic alterations [20].

Quinoa (*Chenopodium quinoa* Willd.) is an Andean grain that is attracting attention worldwide for its nutritional value and as a promising source for the development of functional foods [21,22]. Quinoa grains present a high-quality protein content and an excellent amino acid profile, with higher levels of lysine, methionine, and cysteine than conventional cereals and legumes [23]. Despite being well-known that quinoa consumption provides several benefits for human health [24], special efforts are currently being made to obtain information about the potential effects of quinoa proteins, such as those presenting enzyme inhibitory, antihypertensive, antidiabetic, chemopreventive, and anti-SARS-CoV2 bioactivities [25,26,27,28]. Specifically, with respect to immunonutrition, recent publications have reported the immunomodulation capacity of chenopodin (11S globulin) in intestinal cell models [29], and the immunonutritional impact of 2S albumin quinoa polypeptides and STPIs in adjusting dietary intervention strategies for immunometabolic-based diseases [30,31,32]. However, these studies are focused on experimentally testing the bioactivity of specific quinoa proteins, whereas untargeted studies providing a comprehensive list of quinoa proteins with potential immunonutritional bioactivities have not been described yet. In this regard, only a few publications have reported the use of untargeted proteomics approaches for the identification of quinoa proteins involved in the regulation of oxidative stress signaling pathways, and none of them have been focused on immunonutritional studies [33,34,35].

In this work, a quinoa grain proteome map obtained by label-free mass spectrometry-based shotgun proteomics in our previous study [36] was investigated for the identification of quinoa grain proteins with potential immunonutritional bioactivities, including those related to cancer. The scarce information about the immunonutritional bioactivity of quinoa grain proteins was circumvented by considering their sequence similarity with already described plant-derived AMPs, OSIPs, and STPIs. Similarities and differences at the immunonutritional proteome level between four different commercial quinoa grains, which group different varieties marketed as black (B), red (R), white (from Peru) (W), and royal (white from Bolivia) (RO), were also evaluated before determining the most relevant bioactive proteins. The simple proteomics data mining strategy presented here provides the most comprehensive map of quinoa grain proteins with potential immunonutritional properties existing to date in the literature, and is a starting point for future bioactivity testing of the most promising quinoa grain proteins and commercial classes.

## 2. Materials and Methods

### 2.1. Obtaining FASTA Protein Sequences from Plant-Derived AMPs, OSIPs, and STPIs

FASTA sequences from plant-derived peptides and proteins with immunomodulatory, anti-inflammatory, and anticancer bioactivities, i.e., AMPs, OSIPs, and STPIs, were obtained from the entries collected after a protein source search (filtered by plant organism) with the keywords antimicrobial peptide, oxidative stress induced peptide, and serine-type protease inhibitor in the National Center for Biotechnology Information database (NCBI, https://www.ncbi.nlm.nih.gov/protein, accessed on 1 November 2022). For AMPs, the keywords cycloviolacin O_2_ from *viola odorata*, gamma-thionin from *capsicum chinense*, kalata B1 from *oldenlandia affinis*, NaD1 from *nicotiana alata*, pyrularia from *pyrularia pubera*, and Varv from *viola arvensis* were also included because, despite the fact that they are not returned under the keyword antimicrobial peptide, they are classified as AMPs with recognized antimicrobial, immune-regulatory, and anticancer activities [9]. FASTA protein sequences for AMPs, OSIPs, and STPIs can be found in Supplementary Datafiles S1, S2, and S3, respectively.

### 2.2. Determination of Protein Sequence Similarities

Sequence similarity between the obtained FASTA protein sequences from plant-derived AMPs, OSIPs, and STPIs (Supplementary Datafiles S1, S2, and S3, respectively) and the Reference Sequence (RefSeq) NCBI quinoa database (63,373 protein entries, Supplementary Datafile S4) was calculated using the protein–protein Basic Local Alignment Search Tool (BLASTp) of the NCBI (https://blast.ncbi.nlm.nih.gov/Blast.cgi?PROGRAM=blastp&PAGE_TYPE=BlastSearch&LINK_LOC=blasthome, accessed on 1 November 2022), which allows detection of multiple local alignments between two protein sequences and supplies information for internal sequence matches. BLASTp was applied with “conditional compositional score matrix adjustment”, a matrix adjustment method to compensate for the amino acid composition of sequences [37], and the expected threshold, i.e., the expected number of chance matches in a random model, was chosen as 0.05. Other BLASTp parameters were: scoring alignment matrix BLOSUM62, gap existence 11, gap extension 1, and the options “automatically adjust parameters for short input sequences” and “low complexity regions” enabled (definition of these parameters can be found in https://blast.ncbi.nlm.nih.gov/doc/blast-topics/blastsearchparams.html, accessed on 1 November 2022). Sequence similarity was expressed as percent identity, which determines the matched amino acids (i.e., same residues at the same position) when two sequences are aligned [38]. It is important to note that, for each quinoa protein, only matches with AMPs, OSIPs, and STPIs providing the highest percent identity were selected. After BLASTp analysis, NCBI entries corresponding to quinoa proteins with potential immunonutritional bioactivities were searched against the experimental quinoa grain proteome map from R, B, W, and RO quinoa grains obtained in our previous work (1211 proteins [36]). Additionally, biologically relevant domains in the FASTA protein sequences from plant-derived AMPs, OSIPs, and STPIs were carefully explored with the Conserved Domain Architecture Retrieval Tool (CDART) of the NCBI (https://www.ncbi.nlm.nih.gov/Structure/lexington/lexington.cgi, accessed on 1 November 2022), which finds protein similarities across significant evolutionary distances using sensitive domain profiles rather than sequence similarity. CDART works through the Conserved Domain Database (CDD), which was applied under default parameters with “composition-based statistics adjustment”, an expected threshold of 0.01, and the low complexity filter enabled (definition of these parameters can be found in https://www.ncbi.nlm.nih.gov/Structure/cdd/cdd_help.shtml#WRPSBFilter, accessed on 1 November 2022).

### 2.3. Data Interpretation

Inspection and visualization of the data was performed using Venn diagrams and heat maps. Specifically, graphical representation using Venn diagrams was performed with the Venn diagram R package (version 1.7.3) [39], whereas the freely available web server Heatmappper (http://www.heatmapper.ca) was used for the construction of the heat maps. Finally, in order to find the most relevant bioactive proteins, a scatterplot was constructed by representing, for the identified proteins, their sequence similarity with the original plant-derived bioactive proteins (with a percent identity higher than 65%, *x*-axis) versus their average normalized label-free quantification (LFQ) intensities (*y*-axis).

## 3. Results

### 3.1. Identification of Quinoa Grain Proteins with Immunonutritional Bioactivities

In our previous study [36], proteins from R, B, W, and RO commercial quinoa grains were extracted by alkaline extraction with NaOH followed by isoelectric precipitation at pH 5.0. Then, they were digested with trypsin and analyzed by liquid chromatography coupled to tandem mass spectrometry (LC-MS/MS). After identification and quantification using MaxQuant/Andromeda against the RefSeq NCBI quinoa database, a total of 1211 quinoa grain proteins were identified, with only 21, 30, 88, and 17 being exclusively identified in R, B, W, and RO quinoa, respectively. The complete list of protein group levels, NCBI accession numbers (ID), protein names, Andromeda scores (i.e., identification accuracy), relative molecular masses (M_r_), and average normalized LFQ intensities (n = 3 replicates) for the identified proteins in the different commercial quinoa grains were reported by Galindo-Luján et al. [36]. That data set constitutes the most comprehensive and detailed experimental quinoa grain proteome map existing to date in the literature.

**Figure 1:**
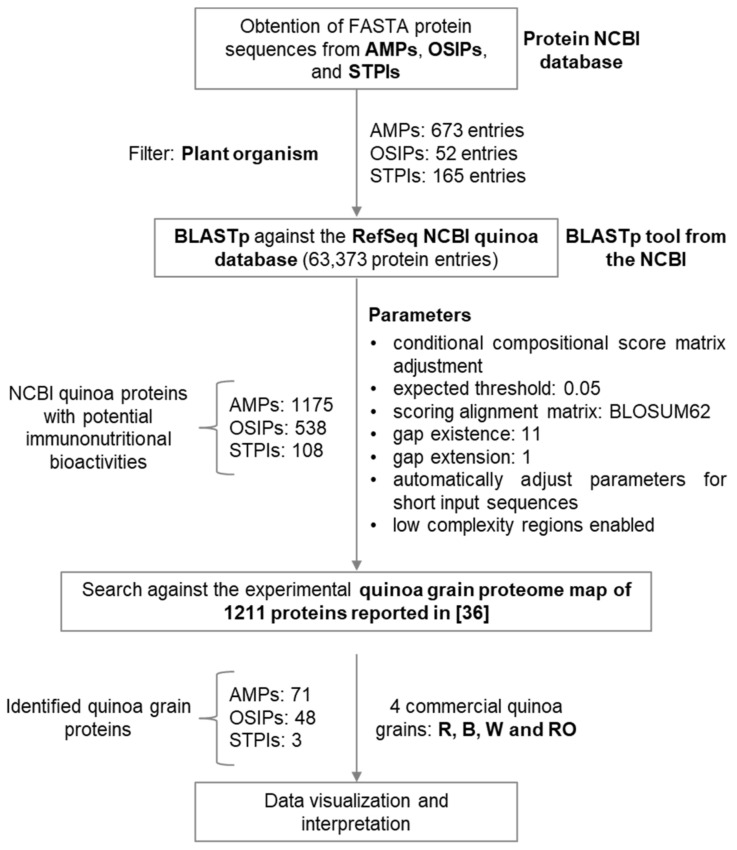
Schematic representation of the proteomics data mining strategy.

In this follow-up study, the quinoa grain proteome map reported in [36] was investigated following the data mining strategy schematized in Figure 1 for the identification of quinoa grain proteins with potential immunonutritional bioactivities, including those related to cancer. As the available information about the immunonutritional bioactivity of quinoa proteins is scarce [29,30,31,32,33,34,35], it was necessary to obtain FASTA protein sequences from plant-derived AMPs (673 entries, Supplementary Table S1), OSIPs (52 entries, Supplementary Table S1), and STPIs (165 entries, Supplementary Table S1), and to perform BLASTp analysis against the RefSeq NCBI quinoa database, which contains 63,373 protein entries. Once sequence similarity (expressed as percent identity) was determined, NCBI entries corresponding to quinoa proteins with potential immunonutritional bioactivities (1175 for AMPs, 538 for OSIPs, and 108 for STIPs) were searched against the experimental quinoa grain proteome map of 1211 proteins identified by proteomics in R, B, W, and RO quinoa grains (reported in [36]). After that, a total number of 71, 48, and 3 quinoa grain proteins were classified as AMPs, OSIPs, and STPIs, respectively, hence suggesting their potential as immunomodulatory, anti-inflammatory, and anticancer agents. In order to complement the results obtained, we carefully checked the biologically relevant domains of the FASTA protein sequences from plant-derived AMPs, OSIPs, and STPIs through the CDART of the NCBI, as we hypothesized that the highest similarity in the domains holding the biological activity would also be relevant to explain the immunonutritional bioactivity of the identified quinoa proteins. Unfortunately, after performing CDART, no matches with the dataset of 1211 quinoa proteins from R, B, W, and RO grains were found. For this reason, and considering that *Chenopodium quinoa* is a non-model plant organism, we can assume that the proposed data mining strategy based exclusively on sequence similarity is at present the best alternative to find relevant quinoa grain proteins for immunonutrition.

Supplementary Tables S2–S4 show the protein group level, the NCBI ID and protein name, the Andromeda score, the M_r_, the average normalized LFQ intensity, and the plant sequence similarity for the 71, 48, and 3 quinoa grain proteins from R, B, W, and RO quinoa that were classified as AMPs, OSIPs, and STPIs, respectively. In these Supplementary Tables, quinoa grain proteins with potential immunonutritional bioactivities were ordered by the Andromeda score, which reflected the identification accuracy by proteomics [36]. As can be observed, the Andromeda score for the identified quinoa grain proteins ranged between 323 and 2 for AMPs, 323 and 3 for OSIPs, and 57 and 3 for STIPs (Supplementary Tables S2–S4, respectively), being more reliable quinoa grain proteins with higher Andromeda scores than those with lower scores. Regarding sequence similarity, identity ranged between 23–100% for AMPs, 23–86% for OSIPs, and 46–49% for SPTIs (Supplementary Tables S2–S4, respectively). In addition, it can also be observed that, in contrast to the AMPs, which presented sequence similarities to AMPs from a wide range of plant organisms, the OSIPs and STPIs were mostly similar to OSIPs and STPIs from *Arabidopsis thaliana*, a widely recognized model plant organism.

**Figure 2:**
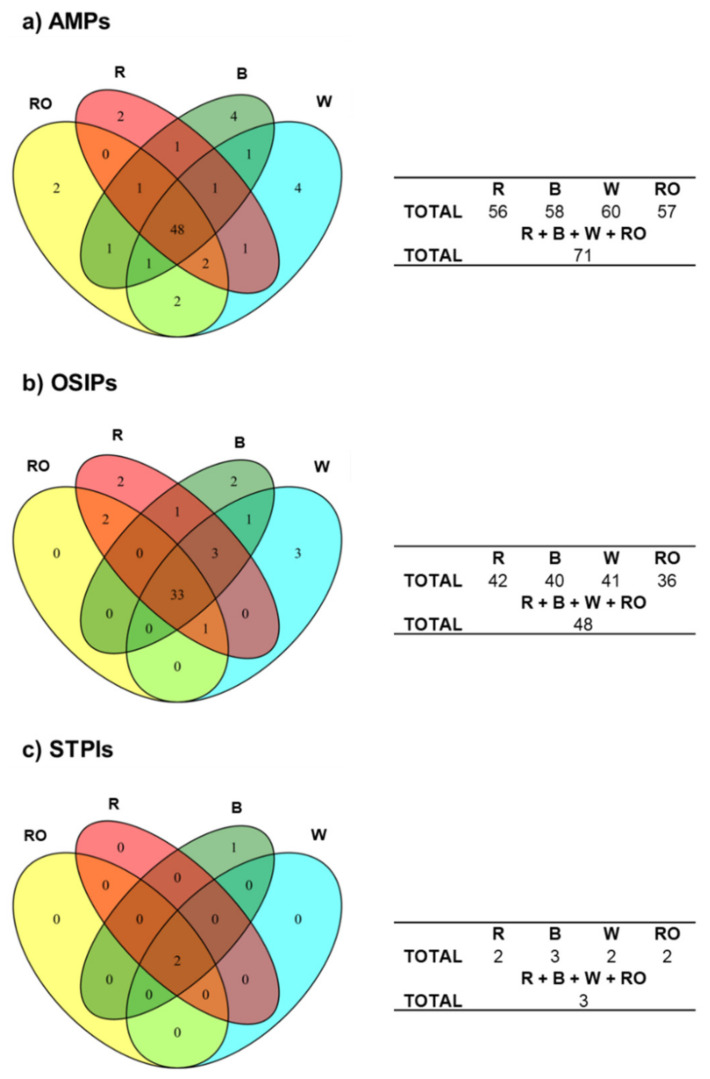
Venn diagrams of the identified (**a**) AMPs, (**b**) OSIPs, and (**c**) STPIs in the four analyzed commercial quinoa grains, R, B, W, and RO.

The Venn diagrams in Figure 2a–c show the relationships between the identified quinoa proteins for R, B, W, and RO grains regarding AMPs, OSIPs, and STPIs, respectively. As can be seen in Figure 2a for AMPs, similar total numbers of proteins were identified as AMPs in R, B, W, and RO quinoa (i.e., 56, 58, 60, and 57, respectively, from the total of 71 AMPs considering the four quinoa grain classes). Among them, 48 AMPs (68% of the total) were identified in all the classes, while 23 (32% of the total) were only present in some of them. Regarding AMPs identified in only one class, 2 were exclusively identified in R, 4 in B, 4 in W, and 2 in RO quinoa. In the case of OSIPs (Figure 2b), similar total numbers of proteins were also identified as OSIPs in R, B, W, and RO quinoa (i.e., 42, 40, 41, and 36, respectively, from the total of 48 OSIPs considering the four quinoa grain classes). Among them, 33 OSIPs (69% of the total) were identified in all the classes, while 15 (31% of the total) were only present in some of them. Regarding OSIPs identified in only one class, 2 were exclusively identified in R, 2 in B, and 3 in W quinoa. Finally, from the total of 3 STPIs (Figure 2c), 2 were identified in R, 3 in B, 2 in W, and 2 in RO quinoa. Among them, 2 STIPs were present in all the quinoa grain classes, while 1 was exclusively identified in B quinoa. All these observations regarding AMPs, OSIPs, and STPIs suggested differences at the immunonutritional proteome level between the four commercial quinoa grains.

**Figure 3:**
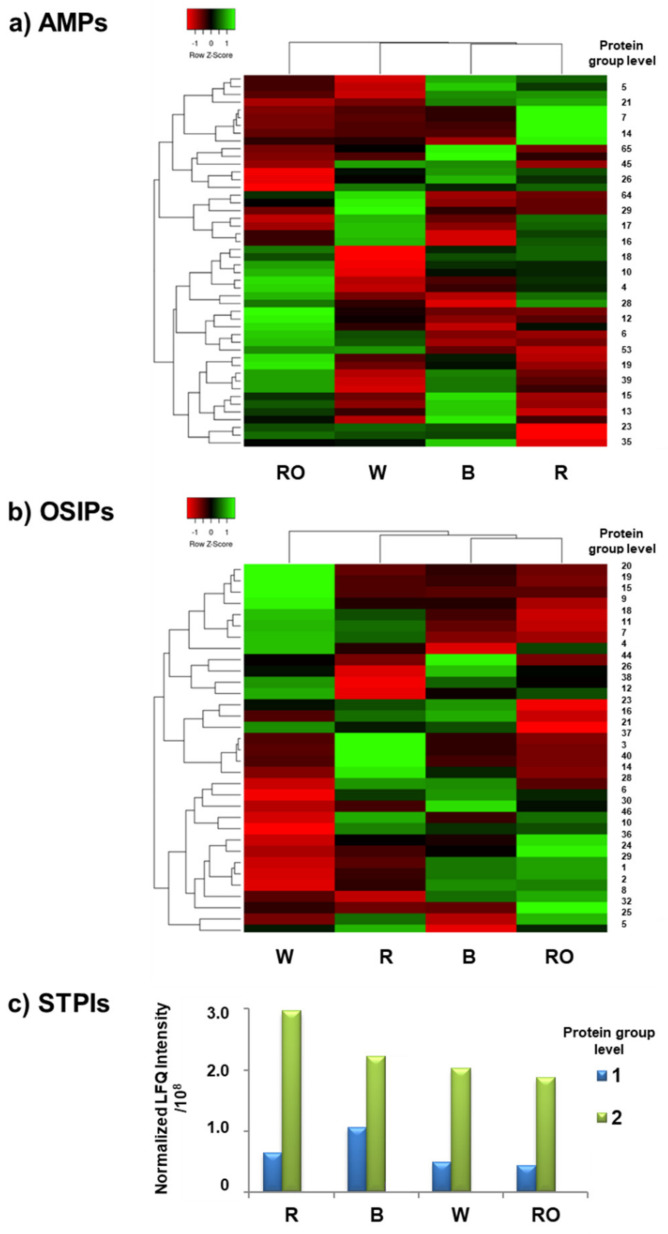
Heat maps obtained using the row z-score normalized LFQ intensities of the identified (**a**) AMPs and (**b**) OSIPs in the four analyzed commercial quinoa grains, R, B, W, and RO; (**c**) bar graph obtained using the average normalized LFQ intensities of the identified STPIs in the four quinoa grains. Only AMPs, OSIPs, and STIPs identified in the four quinoa classes were considered.

Although the Venn diagrams allowed visualization of the general relationships in the number of AMPs, OSIPs, and STPIs identified in the four quinoa grains, it was necessary to consider differences at the concentration level for a more confident discrimination. Euclidean distance heat map graphs were constructed from the data matrix of average normalized LFQ intensities (n = 3, percentage of relative standard deviation (%RSD) < 10% in all cases) of the identified AMPs (Figure 3a) and OSIPs (Figure 3b) in the four quinoa grain classes. Proteins were filtered for complete observations in the four classes (48 out of 71 for AMPs and 33 out of 48 for OSIPs), and z-scores (normalized per protein) were calculated by subtracting the mean and dividing by the standard deviation values. In the heat maps, rows (proteins) and columns (samples) are reordered to keep those with similar profiles closer, with each row z-score entry in the data matrix displayed as a color, making it possible to view the relationships graphically [40]. In addition, they use an agglomerative clustering algorithm to group the data according to the observed characteristic profiles. When two clusters are connected, a line is drawn at a height corresponding to how similar the clusters are. As can be observed in Figure 3a for AMPs, each quinoa grain class presented a characteristic protein concentration profile, with green, red, and black boxes representing up-regulated, down-regulated, and unchanged expression proteins, respectively. As shown in the figure, R and B quinoa grain classes were clustered together, followed by W and, finally, RO quinoa, which, according to the clusters, was the least closely related quinoa grain based on the quantified proteins. Regarding OSIPs (Figure 3b), the protein concentration profile was also characteristic for each quinoa grain, but clustering was different to that obtained for AMPs. In this case, RO and B grain classes were clustered together, followed by R and, finally, W quinoa. Regarding STPIs, as only 2 were identified in the four quinoa grain classes (protein group levels 1 and 2, see Supplementary Table S4), a simple bar graph was presented (Figure 3c), which considers their average normalized LFQ intensities. As can be seen in Figure 3c, the concentration profiles of STPIs for the four quinoa classes were similar, but B and especially R quinoa grains presented higher protein amounts than W and RO quinoa. All these observations suggested differences in the concentration profiles of AMPs, OSIPs, and STPIs between the four commercial quinoa grains, which could be useful to select the quinoa grain class with the protein profile richest in AMPs, OSIPs, and STIPs, and hence with higher immunonutritional bioactivity.

### 3.2. Determination of the Most Relevant Quinoa Grain Proteins with Immunonutritional Bioactivities

**Figure 4:**
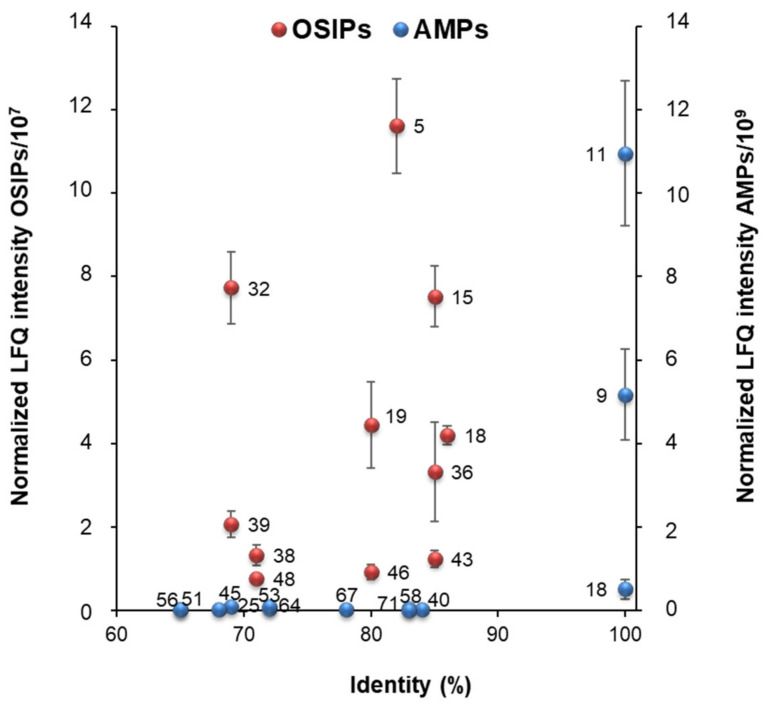
Scatterplot showing the relationship between the sequence similarity of the identified proteins (expressed as percent identity) with the original plant-derived bioactive proteins (*x*-axis) and their normalized LFQ intensities (*y*-axis). Averages of the normalized LFQ intensities obtained for the four analyzed quinoa grains and errors bars for the standard deviation are depicted. A value of 65% identity was set as threshold to increase the significance of the interpretation (13 out of the 71 AMPs, 11 out of the 48 OSIPs, and none of the STPIs were included in the representation).

As the most relevant quinoa grain proteins with immunonutritional bioactivities were supposed to be those with the highest similarity and abundance, the relationship between the sequence similarity of the identified proteins with the original plant-derived bioactive proteins and their normalized LFQ intensities was evaluated. Figure 4 shows a scatterplot where the *x*-axis represents the percent identity (i.e., sequence similarity) and the *y*-axis represents the average normalized LFQ intensities of the quinoa proteins for the four quinoa grain classes (error bars for the standard deviation are depicted in the figure). It is important to note that only AMPs and OSIPs are represented in the scatterplot because an identity threshold of 65% was considered to increase the significance of the interpretation (this value was observed to be the best compromise between the number of immunonutritional proteins and the reliability of the identifications). Setting this elevated threshold, the complete set of identified AMPs and OSIPs was reduced to 13 (out of the 71 AMPs) and 11 (out of the 48 OSIPs). Table 1 shows the protein group level, the NCBI ID, the protein name, the M_r_, and the plant sequence similarity for the 13 AMPs and 11 OSIPs identified with an identity ≥ 65%. In general, as can be observed in Figure 4, normalized LFQ intensities for quinoa AMPs were higher than for OSIPs (LFQ intensities/10^9^ for AMPs vs. LFQ intensities/10^7^ for OSIPs, see the *y*-axis in Figure 4), suggesting that AMPs, due to their higher abundance, could play a more important role in the immunonutritional potential of quinoa grain.

Regarding AMPs (blue spheres in Figure 4), the most relevant bioactive quinoa grain proteins (higher sequence similarity and abundance) would be legumin A-like (protein group levels 11 and 9, Table 1) and antimicrobial peptide 2-like (protein group level 18, Table 1). It is worth mentioning that legumin A-like appears as two independent group levels because their NCBI sequence entries differ in one amino acid (53,642 M_r_ for protein group level 9, and 53,576 M_r_ for protein group level 11, Table 1). Apart from antimicrobial peptide 2-like (12,973 M_r_, Table 1), which belongs to *Chenopodium quinoa* and, therefore, the identity is 100%, legumin A-like presented a 100% identity with defensin-like protein (also known as sesquin) from *vigna unguiculata subsp. sesquipedalis* (ID P84868.1, 1157 M_r_, Table 1) and gymnin from *gymnocladus chinensis* (ID P84200.1, 1171 M_r_, Table 1). These findings are especially interesting, as both plant defensins have been also reported to present immune-regulatory and anticancer activities [9,41,42].

Regarding OSIPs (red spheres in Figure 4), the most relevant bioactive quinoa grain proteins would be putative aconitate hydratase (protein group levels 5 and 18, Table 1), fructose-bisphosphate aldolase 3 (protein group level 15, Table 1), 2-cys peroxiredoxin BAS1 (protein group level 19, Table 1), probable phospholipid hydroperoxide glutathione peroxidase (protein group level 32, Table 1), and peroxiredoxin Q (protein group level 36, Table 1). Again, it is worth mentioning that putative aconitate hydratase appears as two independent group levels because their NCBI sequence entries differ in the number of amino acids (108,650 M_r_ for protein group level 5 and 97,932 M_r_ for protein group level 18, Table 1). As can be seen in Table 1, putative aconitate hydratase group levels 5 and 18 presented 82% and 86% identities, respectively, with aconitate hydratase 3 from *Arabidopsis thaliana* (ID Q9SIB9.2, 108,200 M_r_), fructose-bisphosphate aldolase 3 (43,229 M_r_) presented an 85% identity with fructose-bisphosphate aldolase 3 from *Arabidopsis thaliana* (ID Q9ZU52.1, 42,327 M_r_), 2-cys peroxiredoxin BAS1 (29,900 M_r_) presented an 80% identity with 2-Cys peroxiredoxin BAS1 from *Arabidopsis thaliana* (ID Q96291.2, 29,092 M_r_), probable phospholipid hydroperoxide glutathione peroxidase (26,199 M_r_) presented a 69% identity with probable phospholipid hydroperoxide glutathione peroxidase 6 from *Arabidopsis thaliana* (ID O48646.2, 25,584 M_r_), and peroxiredoxin Q (23,654 M_r_) presented an identity of 85% with peroxiredoxin Q from *Suaeda salsa* (ID Q6UBI3.1, 23,589 M_r_). These findings are also relevant because all these plant-derived OSIPs have been described as playing key roles in regulating resistance to oxidative stress and cell damage [43,44,45,46,47].

## 4. Discussion

The benefits of quinoa consumption for human health have been extensively reported [24], to such an extent that it is being indicated as a promising source for the development of functional foods and nutraceutical products [21,22]. Indeed, it is well-established that quinoa proteins show potential bioactivities toward the promotion of well-being and disease prevention, such as those presenting enzyme inhibitory, antihypertensive, antidiabetic, chemopreventive, and anti-SARS-CoV2 bioactivities [25,26,27,28]. In immunonutrition, a recent publication has reported the capacity of chenopodin (11S globulin), the major protein component of quinoa grains, to regulate immune-mediated pathways in a human intestinal cell model following the trigger of inflammation [29]. This agrees with the results described in our study, where we identified a quinoa grain protein from the 11S globulin family, i.e., 11S globulin seed storage protein 2-like (protein group levels 10, 27, and 42, Supplementary Table S2), as an AMP. Other publications have also described the immunonutritional impact of 2S albumin quinoa polypeptides and STPIs (not found in our work) to regulate dietary strategies in immune-mediated diseases, including obesity and hepatocarcinoma [30,31,32]. However, these studies are focused on performing experimental tests to evaluate the bioactivity of specific quinoa proteins, whereas untargeted studies providing a complete list of quinoa proteins with immunonutritional bioactivities are not described yet.

After carefully revising the existing literature, only three publications were found that describe untargeted proteomics strategies for the identification of quinoa proteins involved in the regulation of oxidative stress signaling pathways, but none of them focused on immunonutrition [33,34,35]. All these studies were based on describing responses at the proteome level, i.e., up-regulation, down-regulation, and unchanged expression of proteins, after subjecting quinoa plants to infection by mitovirus [33] and salinity treatments [34,35]. In addition, proteomics experiments have been performed on quinoa proteins extracted from leaves [33,34] and guard cells [35]. From the wide variety of quinoa proteins identified in these works [33,34,35], only glycine-rich RNA-binding abscisic acid-inducible protein-like (protein group levels 24 and 13, Supplementary Tables S2 and S3, respectively) was identified in our work as both AMP and OSIP. Glycine-rich RNA-binding abscisic acid-inducible protein-like belongs to the family of glycine-rich proteins, which have glycine contents up to 60–70% and their synthesis is part of the plant’s defense mechanism, suggesting their primary role as AMPs [48]. However, the low number of matching proteins between our work and the previously reported ones stems from the fact that our study is focused on the identification of proteins from quinoa grains (in contrast to leaves and guard cells), hence showing a different proteomic profile.

In contrast to the aforementioned works [33,34,35], the present study intends to have a high impact in immunonutrition, as it is based on the identification of proteins from an edible part of the plant, i.e., grains. Indeed, to the best of our knowledge, it provides the most comprehensive map of quinoa grain proteins with potential immunonutritional properties existing to date in the literature, i.e., 71 AMPs, 48 OSIPs, and 3 STPIs, which present in different proteomic profiles across the four most abundant commercial classes, i.e., R, B, W, and RO (as suggested by the Venn diagrams and heat maps). The presented list could be used as a starting point for future bioactivity testing of the most promising quinoa grain proteins. e.g., those with higher sequence similarity and abundance. Among them, we could highlight the relevance of some OSIPs such as aconitate hydratase (protein group levels 5 and 18, Table 1), fructose-bisphosphate aldolase 3 (protein group level 15, Table 1), 2-cys peroxiredoxin BAS1 (protein group level 19, Table 1), probable phospholipid hydroperoxide glutathione peroxidase (protein group level 32, Table 1), peroxiredoxin Q (protein group level 36, Table 1), and especially the AMP legumin A-like (protein group levels 9 and 11, Table 1). This quinoa protein presents the highest abundance and a 100% identity to the plant defensins sesquin and gymnin, which have been extensively reported to present immune-regulatory and anticancer activities [9,41,42]. However, it is also important to note that only a specific part of the legumin A-like protein sequence is similar to sesquin and gymnin (M_r_ around 53,000 for legumin A-like protein vs. M_r_ around 1000 for sesquin and gymnin), suggesting that the peptides with immunonutritional bioactivities would be obtained after protein hydrolysis, e.g., gastrointestinal digestion. In this sense, in addition to experimental bioactivity tests, complementary bioavailability studies would be needed in order to confirm if these peptides can resist the action of digestive enzymes, cross the intestinal epithelial barrier, and reach the target organs intact where they would exert their health-promoting effects.

## 5. Conclusions

This simple proteomics data mining strategy based on sequence BLASTp analysis allowed classification of 71, 48, and 3 of the identified quinoa grain proteins as AMPs, OSIPs, and STPIs, respectively. Similarities and differences were found at the immunonutritional proteome level between the different quinoa grain classes, and a final set of 13 AMPs and 11 OSIPs were considered to be the most relevant for the immunonutritional potential of quinoa grain. Among them, the legumin A-like, which presented the highest abundance and a 100% identity to already described AMPs, the plant defensins sesquin and gymnin, was highlighted. The presented strategy allowed the creation of the most comprehensive map of quinoa grain proteins with potential immunonutritional properties existing to date in the literature, as a starting point for future bioactivity and bioavailability testing of the most promising quinoa grain proteins and commercial classes. Furthermore, the proteomics data mining strategy presented in this study can be adapted to target other interesting bioactivities in quinoa grains or even other edible non-model organisms.

## Figures and Tables

**Table 1:** Protein group level, NCBI accession number (ID), protein name, relative molecular mass (M_r_), and plant sequence similarity for the 13 AMPs and 11 OSIPs identified with an identity ≥ 65% (threshold selected to increase the significance of the interpretation).

*Chenopodium quinoa*				Plant Sequence Similarity				
^1^ Protein Group Level	ID	Protein Name	M_r_	Plant Organism	ID	Protein Name	M_r_	^2^ Identity (%)
AMPs								
9	XP_021768828.1	legumin A-like	53,642	*Vigna unguiculata subsp. sesquipedalis*	P84868.1	defensin-like protein	1157	100
				*Gymnocladus chinensis*	P84200.1	gymnin	1171	
11	XP_021770181.1	legumin A-like	53,576	*Vigna unguiculata subsp. sesquipedalis*	P84868.1	defensin-like protein	1157	100
				*Gymnocladus chinensis*	P84200.1	gymnin	1171	
18	XP_021717953.1	antimicrobial peptide 2-like	12,973	*Chenopodium quinoa*	XP_021717953.1	antimicrobial peptide 2-like	12,973	100
25	XP_021746256.1	protein disulfide isomerase-like 1–4	64,057	*Oldenlandia affinis*	ABS11216.1	protein disulfide isomerase precursor, partial	58,968	69
40	XP_021768967.1	caffeine synthase 1-like	43,964	*Vigna unguiculata subsp. sesquipedalis*	P84868.1	defensin-like protein	1157	83
45	XP_021728344.1	protein disulfide isomerase-like 1–4	64,758	*Oldenlandia affinis*	ABS11216.1	protein disulfide isomerase precursor, partial	58,968	68
51	XP_021760599.1; XP_021725721.1	defensin-like protein 2; defensin Ec-AMP-D2-like	8588	*Solanum lycopersicum var. Cerasiforme*	ADK36631.1	defensin-like protein, partial	5351	69
53	XP_021714401.1; XP_021735018.1	peamaclein-like	10,270	*Solanum tuberosum*	AAD01518.1	snakin-1, partial	5491	72
56	XP_021749254.1	peamaclein-like	10,287	*Solanum tuberosum*	AAD01518.1	snakin-1, partial	5491	65
58	XP_021771951.1; XP_021771949.1; XP_021728837.1	serine/threonine-protein phosphatase PP2A-2 catalytic subunit isoform X2; isoform X1; subunit-like	30,928	*Peltophorum dubium*	AWY94151.1	protein phosphatase 2A, partial	10,719	84
64	XP_021735074.1; XP_021714402.1	peamaclein-like	10,322	*Solanum tuberosum*	AAD01518.1	snakin-1, partial	5491	72
67	XP_021714548.1; XP_021718041.1	gamma carbonic anhydrase 1, mitochondrial-like	28,557	*Viola odorata*	P58434.1	cycloviolacin-O2	3165	78
				*Viola odorata*	2KNM_A	chain A, cycloviolacin-O2	3165	
				*Viola odorata*	2KCG_A	chain A, cycloviolacin-O2	3165	
71	XP_021773131.1; XP_021742272.1	probable prefoldin subunit 4	14,794	*Vigna unguiculata subsp. sesquipedalis*	P84868.1	defensin-like protein	1157	83
				*Gymnocladus chinensis*	P84200.1	gymnin	1171	
OSIPs								
5	XP_021774578.1	putative aconitate hydratase, cytoplasmic	108,650	*Arabidopsis thaliana*	Q9SIB9.2	aconitate hydratase 3	108,200	82
15	XP_021764772.1	fructose-bisphosphate aldolase 3, chloroplastic	43,229	*Arabidopsis thaliana*	Q9ZU52.1	fructose-bisphosphate aldolase 3	42,327	85
18	XP_021774583.1	putative aconitate hydratase, cytoplasmic	97,932	*Arabidopsis thaliana*	Q9SIB9.2	aconitate hydratase 3	108,200	86
19	XP_021765715.1; XP_021766570.1; XP_021765707.1	2-Cys peroxiredoxin BAS1, chloroplastic-like isoform X2; chloroplastic; chloroplastic-like isoform X1	29,900	*Arabidopsis thaliana*	Q96291.2	2-Cys peroxiredoxin BAS1	29,092	80
32	XP_021754079.1	probable phospholipid hydroperoxide glutathione peroxidase	26,199	*Arabidopsis thaliana*	O48646.2	probable phospholipid hydroperoxide glutathione peroxidase 6	25,584	69
36	XP_021756717.1	peroxiredoxin Q, chloroplastic-like	23,654	*Suaeda salsa*	Q6UBI3.1	peroxiredoxin Q	23,589	85
38	XP_021746311.1; XP_021746313.1	probable phospholipid hydroperoxide glutathione peroxidase isoform X1; isoform X2	26,468	*Arabidopsis thaliana*	O22850.1	probable glutathione peroxidase 3	23,258	71
39	XP_021773039.1	superoxide dismutase [Cu-Zn]	15,262	*Arabidopsis thaliana*	O78310.2	superoxide dismutase [Cu-Zn] 2	22,244	69
43	XP_021728703.1	fructose-bisphosphate aldolase 3, chloroplastic-like	43,043	*Arabidopsis thaliana*	Q9ZU52.1	fructose-bisphosphate aldolase 3	42,327	85
46	XP_021776210.1; XP_021717201.1	peptide methionine sulfoxide reductase B5-like	24,740	*Arabidopsis thaliana*	Q9M0Z6.2	peptide methionine sulfoxide reductase B3	18,847	80
48	XP_021770821.1; XP_021770820.1	acylpyruvase FAHD1, mitochondrial-like isoform X2; isoform X1	22,257	*Arabidopsis thaliana*	Q93ZE5.1	probable acylpyruvase FAHD1	24,283	71

^1^ Protein group level: there are groups of proteins that are sufficiently similar that the individual proteins cannot be distinguished based on their peptide content. In MaxQuant, identification and quantification are reported at the group level. ^2^ Identity (%): determines the matched amino acids (i.e., same residues at the same position) when two sequences are aligned.

## Data Availability

The data are contained within the article and supplementary materials, or will be made available on reasonable request.

## Supplementary Materials

The following supporting information can be downloaded at: https://www.mdpi.com/article/10.3390/foods12020390/s1, Table S1: Entry number, NCBI accession number (ID), GI accession, protein name, plant organism, number of amino acids, and entry type for the plant-derived AMPs (673 entries), OSIPs (52 entries), and STPIs (165 entries) found in the NCBI database; Table S2: Protein group level, NCBI accession number (ID) and protein name, Andromeda score, relative molecular mass (M_r_), average normalized LFQ intensity, and plant sequence similarity for the 71 quinoa grain proteins from R, B, W, and RO quinoa that were classified as AMPs; Table S3: Protein group level, NCBI accession number (ID) and protein name, Andromeda score, relative molecular mass (M_r_), average normalized LFQ intensity, and plant sequence similarity for the 48 quinoa grain proteins from R, B, W, and RO quinoa that were classified as OSIPs; Table S4: Protein group level, NCBI accession number (ID) and protein name, Andromeda score, relative molecular mass (M_r_), average normalized LFQ intensity, and plant sequence similarity for the 3 quinoa grain proteins from R, B, W, and RO quinoa that were classified as STPIs; Datafile S1: FASTA protein sequences for the 673 entries from plant-derived AMPs found in the NCBI database; Datafile S2: FASTA protein sequences for the 52 entries from plant-derived OSIPs found in the NCBI database; Datafile S3: FASTA protein sequences for the 165 entries from plant-derived STPIs found in the NCBI database; Datafile S4: FASTA protein sequences for the 63,373 entries found in the RefSeq NCBI quinoa database.
